# Preparation, Physicochemical Properties and Hemocompatibility of Biodegradable Chitooligosaccharide-Based Polyurethane

**DOI:** 10.3390/polym10060580

**Published:** 2018-05-24

**Authors:** Weiwei Xu, Minghui Xiao, Litong Yuan, Jun Zhang, Zhaosheng Hou

**Affiliations:** College of Chemistry, Chemical Engineering and Materials Science, Shandong Normal University, Jinan 250014, China; 17853135516@163.com (W.X.); xiaominghui98@163.com (M.X.); m17853135556@163.com (L.Y.); zj971127@163.com (J.Z.)

**Keywords:** chitooligosaccharide, polyurethane, biodegradability, physicochemical properties, hemocompatibility

## Abstract

The purpose of this study was to develop a process to achieve biodegradable chitooligosaccharide-based polyurethane (CPU) with improved hemocompatibility and mechanical properties. A series of CPUs with varying chitooligosaccharide (COS) content were prepared according to the conventional two-step method. First, the prepolymer was synthesized from poly(ε-caprolactone) (PCL) and uniform-size diurethane diisocyanates (HBH). Then, the prepolymer was chain-extended by COS in *N*,*N*-dimethylformamide (DMF) to obtain the weak-crosslinked CPU, and the corresponding films were obtained from the DMF solution by the solvent evaporation method. The uniform-size hard segments and slight crosslinking of CPU were beneficial for enhancing the mechanical properties, which were one of the essential requirements for long-term implant biomaterials. The chemical structure was characterized by FT-IR, and the influence of COS content in CPU on the physicochemical properties and hemocompatibility was extensively researched. The thermal stability studies indicated that the CPU films had lower initial decomposition temperature and higher maximum decomposition temperature than pure polyurethane (CPU-1.0) film. The ultimate stress, initial modulus, and surface hydrophilicity increased with the increment of COS content, while the strain at break and water absorption decreased, which was due to the increment of crosslinking density. The results of in vitro degradation signified that the degradation rate increased with the increasing content of COS in CPU, demonstrating that the degradation rate could be controlled by adjusting COS content. The surface hemocompatibility was examined by protein adsorption and platelet adhesion tests. It was found that the CPU films had improved resistance to protein adsorption and possessed good resistance to platelet adhesion. The slow degradation rate and good hemocompatibility of the CPUs showed great potential in blood-contacting devices. In addition, many active amino and hydroxyl groups contained in the structure of CPU could carry out further modification, which made it an excellent candidate for wide application in biomedical field.

## 1. Introduction

Polyurethanes (PUs) are becoming more and more important as engineering materials because of their excellent abrasion resistance and the properties of both rubber and plastics [1,2,3]. The hard-segment-rich and soft-segment-rich domains existing in PUs contribute to the specific microphase-separated structure, which give them unique mechanical properties [4]. Due to the excellent physic-mechanical properties and good biocompatibility, PUs have been used in many biomedical engineering areas, including blood vessels, cardioids, artificial skins, cartilages, joints, and catheters [5,6,7]. But, when PUs are used as long-term blood-contacting materials, surface-induced thrombosis, protein fouling, and poor cytocompatibility are three popular problems which are difficult to conquer [8,9]. To achieve improved biocompatibility of synthetic polymers, natural biopolymers were used to prepare novel hemocompatible biocomposites [10,11]. Much attention has been paid to producing a nonspecific protein repelling surface by surface modification via various kinds of methods and creating highly effective non-thrombogenic devices. A preferred strategy is to immobilize natural biopolymers that shield the surface, thus introducing a high activation barrier to repel proteins [12,13,14,15]. But the biopolymers can fall out slowly from the surface in the uage process, which limits the application as long-term implant materials. Currently, much effort has been applied to use natural material in the design and preparation of new biomaterial, and bulk-modified PU by natural biopolymers has become a new frontier [16,17]. Among the natural biopolymers, polysaccharides, which are readily available, inexpensive, and biodegradable, appear to be good candidates for this purpose. 

Chitosan (CH), the linear cationic (1,4)-2-amino-2-deoxy-β-d-glucan produced from the natural parent chitin by partial deacetylation, is the second most abundant polysaccharide in nature and has been utilized in the biomedical field due to its biological properties, such as nontoxicity, thermal stability, cytocompatibility, and hemocompatibility [18]. Several researches have been reported regarding chitosan-polyurethane copolymers [19,20,21], which possess improved thermal stability and mechanical properties. In some of their works, the swollen CH, which is obtained by dispersion in glacial acetic acid/*N*,*N*-dimethylformamide (DMF) mixtures [22], is used as an extender in the copolymerization because CH cannot be dissolved in organic solvent; others use the waterborne PU (WPU) to react with CH in glacial acetic acid [23,24]. However, the CH-modified PU materials obtained by these methods are limited by the processing difficulties, because the forms of these materials are either gel or solid. On the other hand, CH exhibits other drawbacks of poor flexibility and degradability, which are related to chemical and physical characteristics such as the high molecular weight and crystallinity [25]. 

As depolymerized product by chemical and enzymatic hydrolysis of CH, chitooligosaccharide (COS) consisting of 2–10 glucosamine units bounded via β-1,4-glycoside linkages has attracted more and more attention recently, because the latter has a shorter chain length and can be easily soluble in water and in some organic solvent, such as dimethylsulfoxide (DMSO) and DMF. In addition, there are several papers reported that COS can be absorbed readily through the intestine, quickly getting into the blood flow [26,27]. On the other hand, the reactive amino and hydroxyl groups of COS make it easy to prepare COS-based modified biomaterials. Because of its unusual properties, COS shows great potential to be a biopolymer to improve the performance of PUs [28,29,30,31]. Base on the good water solubility of COS, Jia groups prepared novel hemocompatible WPUs using COS as an extender via the self-emulsion polymerization method [17,32]. The COS-based WPU emulsion showed satisfactory freeze/thaw stability, and the films cast from the emulsions exhibited excellent mechanical properties and good anticoagulating character. However, only a little work has been published to describe the synthesis of COS-based PU in organic solvent [33], which can provide a novel way to design and prepare bulk-modified PU biomaterials by COS and exploit the application of PU in biomedical field. 

In this article, a series of block COS-based PUs (CPU) with varying COS content were prepared in organic solvent via the conventional two-step method. First, the prepolymer was synthesized from poly(ε-caprolactone) (PCL) and uniform-size diurethane diisocyanates (HBH). Then, the prepolymer was chain-extended by COS in DMF to obtain the CPU with low crosslinking density, and the corresponding films were prepared from the DMF solution by the solvent evaporation method. The uniform-size hard segments and slight crosslinking of CPU are beneficial for enhancing the mechanical properties, which are the essential requirement for long-term implant biomaterials. The influence of COS content in CPU on the physicochemical properties of the films, including thermal properties, mechanical properties, surface hydrophilicity, swellability, and in vitro hydrolytic biodegradability, were researched. Surface hemocompatibility of the films was examined by protein adsorption and platelet adhesion tests. Moreover, many active amino and hydroxyl groups contained in the structure of CPU could carry out further modification, which made it an excellent candidate for wide application in biomedical field.

## 2. Materials and Methods

### 2.1. Materials

COS (number-average molecular weight: 3000 g/mol; degree of deacetylation: 92%) was supplied by Qingdao Yunzhou Biochemistry Co., Ltd. (Qingdao, China) and dried for 4 h at 50 °C under vacuum prior to use. PCL (number-average molecular weight: 2000 g/mol) was obtained from Shenzhen Polymtek Biomaterial Co., Ltd. (Shenzhen, China) and used without further purification. 1,6-Hexanediisocyanate (HDI) and dibutyltin dilaurate (DBTDL) were purchased from Sigma-Aldrich Chemical Co. (St. Louis, MO, USA) and used as received. 1,4-Butanediol (BDO, Aladdin Reagent Co., Shanghai, China) were dried over 4Å molecular sieves and redistilled before use. DMF (AR grade, Aladdin Reagent Co., Shanghai, China) was dried with phosphorus pentoxide and distilled under reduced pressure prior to use. Phosphate buffer saline (PBS, pH = 7.4) was supplied by Beijing Chemical Reagent Co., Ltd. (Beijing, China) and used as received. Other reagents were AR grade and purified by standard methods. 

### 2.2. Synthesis of CPU

**Figure 1:**
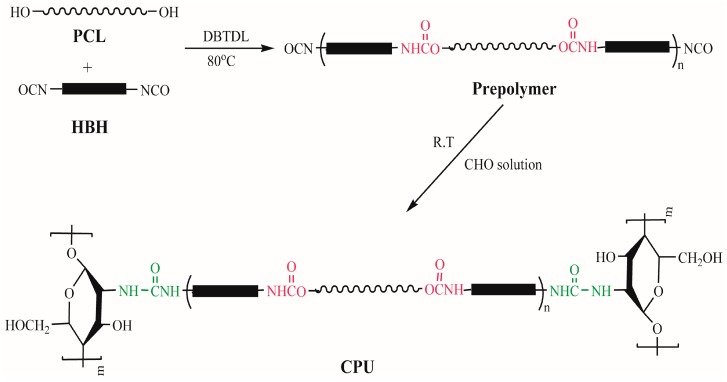
General reaction scheme of CPU composites.

The basic formulations are given in Table 1. A typical procedure was described as below: A predetermined amount of diurethane diisocyanates (1,6-hexanediisocyanate-1,4-butanediol-1,6-hexanediisocyanate, HBH), which was synthesized according to the previous literature [34], and PCL was charged into a three-necked flask equipped with a mechanical stirrer under dried nitrogen atmosphere. DMF was added and the mixture was stirred at room temperature to get a homogeneous solution (~0.4 g/mL). After two drops of DBTDL was added to the solution (0.3 wt % of PCL and HBH), the reaction was carried out at 80 °C for about 2.5 h until the isocyanate group content in the system reaching the theoretical value, which was determined using the standard di-n-butylamine back titration method [35,36]. Then the system (prepolymer solution) was cooled to 25 °C, and the COS solution (DMF, 0.2 g/mL) was added in one portion under vigorous mechanical stirring. When the reaction mixture became viscous, small amounts of DMF were re-added to keep the system homogeneous. The reaction mixture was allowed to proceed at 25 °C for about 1.5 h until the NCO peak (~2270 cm^−1^) in the FT-IR spectrum disappeared to obtain the CPU solution. The reaction was carried out according to the general reaction scheme as shown in Figure 1, and the CPUs was named as CPU-X (X: the molar ratio of *n*_–NCO_:*n*_–OH_).

### 2.3. Preparation of CPU Films

The CPU solution was first diluted with DMF to ~4.5 g/100 mL, and then the diluted solution was cast into a Teflon mold. Most of solvent was first removed by natural volatilizing at 50 °C for four days, and then the last traces of solvent was removed under reduced pressure for one day to obtain the semitransparent pale-brown films with 0.40 ± 0.02 mm thickness. Finally the films were punched into discs with ~10 mm in diameter for measurement.

### 2.4. Characterization

FT-IR: the Fourier transform infrared (FT-IR) spectrophotometer used was an Alpha infrared spectrometer (Bruker, Rheinstetten, Germany) equipped with a Bruker platinum ATR accessory at room temperature. The spectra covered the infrared region 4000–400 cm^−1^ with the resolution of 4 cm^−1^. The scans were collected on COS powder, prepolymer, and CPU films. 

Thermal properties: A differential scanning calorimeter (DSC) (DSC2910, Universal, New Brunswick, NJ, USA) was employed to study the thermal transition behavior of the polymers. The samples (1~1.3 mg) were first heated up to 150 °C at a heating rate of 10 °C/min to erase the thermal history, then cooled to −70 °C at 5 °C/min, and finally heated to 150 °C at 10 °C/min. The measurements were taken under a continuous nitrogen purge (30 mL/min), and the reported thermal transition temperatures were collected during the second heating cycle. Thermogravimetric analysis (TGA) was recorded on a TGA 2050 analyzer (Universal, New Brunswick, NJ, USA). The instrument was calibrated using a pure calcium oxalate sample before analysis. About 8~10 mg of sample was subjected to TGA scans, which were performed from ambient to 800 °C with a heating rate of 20 °C/min under nitrogen atmosphere with a gas flow rate of 50 mL/min. 

Crystallization behavior: The crystallization behaviors of CPU films were measured by X-ray powder diffraction (XRD) analysis. The measurements were conducted by a Max 2200PC power X-ray diffractometer (Rigaku Corp., Tokyo, Japan) with 40 kV and 20 mA using Cu Kα (1.54051 Å) radiation. The sample holder containing samples were scanned from 5° to 55° with a scanning rate of 2*θ* = 0.02°.

Mechanical properties: Tensile strength tests were carried out using a single-column tensile test machine (Model HY939C, Dongguan Hengyu, Ltd., Dongguan, China) at room temperature with a crosshead speed of 50 mm/min. The films were punched into Dumbbell-shaped specimens using a punch cutter with a punching die of 12 mm width and 75 mm length, the neck width and length were 4.0 and 30 mm, respectively. Property values reported here represent averaged results of at least five samples.

Water contact angle: The contact angle measurement was used to evaluate the surface hydrophilicity of the films. The sessile static water contact angle measurements were carried on a drop shape analysis system (CAM 200, KSV Instruments, Helsinki, Finland). The ultrapure water was dripped onto the sample surface at room temperature, and in oder to ensure that the droplet did not soak into the compact, the tests were performed within 10 s. Three different points were tested for each sample and six readings were averaged for each film.

Water absorption: The amount of water that each film absorbed was adopted to quantify the swellability of the CPU films. Each film disc (~10 mm diameter) was immersed in 10 mL deionized water which maintained the temperature of 37 ± 0.1 °C until reaching the water absorption equilibrium (~48 h). Then the discs were removed from water, and the surplus surface water was gently wiped off with a filter paper and weighed. The sample mass change resulting from the water uptake expressed in percent was calculated according to the formula: Water absorption (%) = (*W*_t_ − *W*_o_)/*W*_o_ × 100, where *W*_o_ and *W*_t_ are the weights of dry and wet samples, respectively. Each sample was tested at least five times and the results were averaged.

In vitro degradation: In vitro degradation studies of the films were performed in PBS (pH = 7.4) through the weight loss. The film discs (~10 mm diameter) were placed into an individual sealed bottle containing 10 mL PBS in a biochemical incubator at 37 ± 0.1 °C. At given time intervals, the samples were removed from the buffer, rinsed three times with distilled water and dried to a constant weight at 35 °C under vacuum. Post-degradation weight was measured and mass loss of the films was obtained using the following formula: Weight loss ratio (%) = (*W*_o_ − *W*_r_)/*W*_o_ × 100, where *W*_o_ and *W*_r_ mean the original dry weight and the rest dry weight after degradation for a predetermined time, respectively. The assessments were conducted for 12 months or until the films lost mechanical properties and became fragments. Three samples were tested, and the average value was taken. 

Surface morphologies: A cold field emission scanning electron microscope (FE-SEM, Hitachi SU8010, Tokyo, Japan) was used to investigate the surface morphologies of the films after degradation for a fixed time. The dried discs, which were first mounted on aluminum stubs with conductive graphite-filled tapes, were coated with a gold layer under vacuum and then used for SEM observation.

Protein adsorption: A Bradford protein assay with bovine serum albumin (BSA) as the model protein was used to measure the amount of albumin adsorbed onto the surface. In order to achieve complete hydration, the film discs (~10 mm diameter) were immersed into PBS (pH = 7.4) for about 24 h until equilibrating, and then were filled with 1.0 mL of BSA solution (45 μg/mL, the same as the concentration of normal plasma) at 37 °C for 1 h. After absorption, the discs were taken out and first rinsed with PBS for three times to remove the unbound BSA. Then the adsorbed protein on the surface was desorbed with sodium dodecylsulfonate aqueous solutions (1 wt %) at 37 °C with agitating at 100 rpm for 1 h. A micro-Bradford protein assay kit (Sangon Biotech Co., Ltd., Shanghai, China) with a multiwell microplate reader (Multiskan Mk3-Thermolabsystems, Thermo Fisher Scientific, Inc., Waltham, MA, USA) was used to test the concentration of the adsorbed BSA at 595 nm. The final adsorbed protein quantity could be obtained referring to the standard curve of optical density against BSA concentration. Three independent measurements were performed, and values relative to the control (PBS) were collected.

Platelet adhesion: The interaction between the blood and film surface was assayed by platelet adhesion experiment. Platelet-rich plasma (PRP) was obtained from the fresh rabbit blood (Shandong Success Biological Technology Co., Ltd., Qingzhou, China) containing sodium citrate as an anticoagulant by centrifugation from blood at 2000 rpm for 20 min at 4 °C. The film discs (~10 mm diameter) were first equilibrated with PBS (pH = 7.4) for 12 h, and then were taken out and incubated with 1.0 mL PRP which was pre-warmed to 37 °C. After incubation for 2 h at 37 °C, the discs were rinsed three times with PBS by mild shaking to remove nonadherent and weakly adhered platelets. The platelets adhering to the surface were fixed with a glutaraldehyde solution (2.5 *v*/*v* %) in PBS buffer for 30 min at room temperature. After thorough washing with PBS, the discs were dehydrated by treating with gradual ethanol/water solution from 50% to 100% ethanol (*v*:*v*) with a step of 10% for 30 min in each step and allowed to dry on a clean bench at room temperature. The platelet-attached surfaces were coated with gold prior to this, and different fields were randomly observed by FE-SEM.

## 3. Results and Discussion

### 3.1. FT-IR

**Figure 2:**
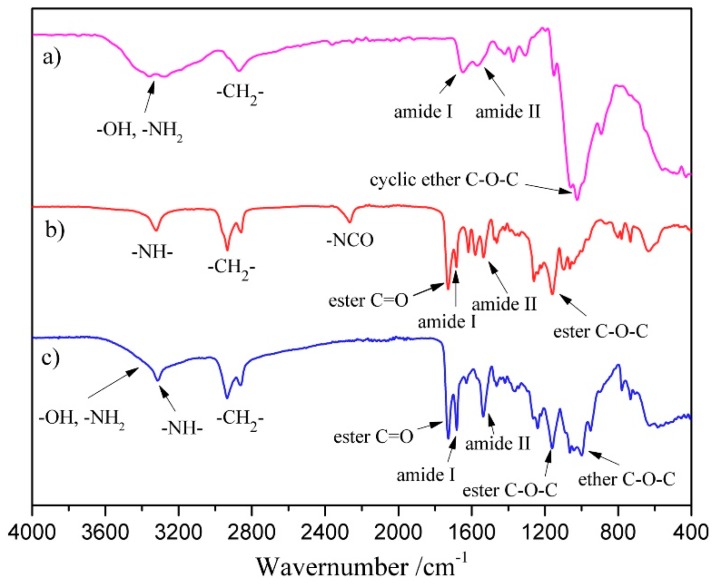
FT-IR spectra of (**a**) COS; (**b**) prepolymer and (**c**) CPU-1.7.

FT-IR spectroscopy has been extensively used in PU research, since it presents an easy method of obtaining direct information on chemical changes. The FT-IR spectra of COS, prepolymer, and CPU-1.7 are shown in Figure 2 (CPUs with different COS content have the similar spectra except for the intensity of the peak). In the spectrum of COS powder (Figure 2a), the broad characteristic peak at about 3200~3420 cm^−1^ was attributed to the stretching vibrations of –OH and –NH_2_, and the absorption peaks at 2882 and 1035 cm^−1^ corresponded to the saturated stretching of –CH_2_ and cyclic ether C–O–C, respectively [37]. The weak absorption bands observed at 1664 and 1549 cm^−1^ belonged to the characteristic absorption peaks of amide I and amide II, which was attributed to the residual amide linkage in COS. In the spectrum of prepolymer (Figure 2b), an obvious absorption peak at 2265 cm^−1^ belonged to the characteristic absorption of terminal –NCO of the prepolymer. The absorbance in the region near 3325 cm^−1^ indicated that most of the N–H groups are hydrogen bonded [38]. The other absorption bands at 1726, 1673, 1535, and 1150 cm^−1^ were attributed to the characteristic stretching frequencies of ester C=O, amide I, amide II, and ester C–O–C, respectively. The absorption peak of –NCO disappeared completely in the spectrum of CPU-1.7 (Figure 2c), meaning that the prepolymer was chain-extended with COS. As the paper reported [18], the ureido will be formed because the –NH_2_ groups have much higher reactivity with –NCO than –OH groups in COS. Meanwhile, the relative intensity of –NH– (~3322 cm^−1^), amide I (~1680 cm^−1^), and amide II (~1535 cm^−1^) increased obviously compared with that of ester C=O, which should be due to the formation of new ureido between COS and the prepolymer. In addition, all the other characteristic absorptions of COS and prepolymers also appeared in the spectrum. The results from FT-IR spectra show the successful preparation of CPU. 

### 3.2. Thermal Transition

**Figure 3:**
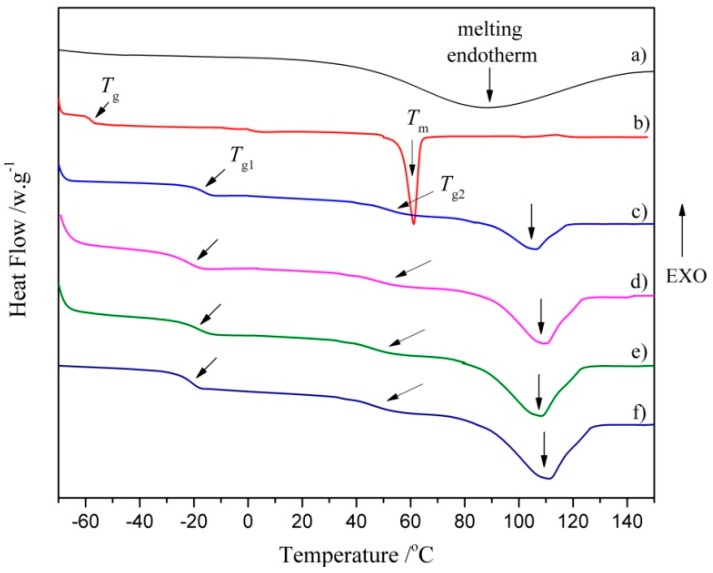
Differential scanning calorimeter DSC thermograms of (**a**) COS; (**b**) poly(ε-caprolactone) PCL; (**c**) CPU-1.0; (**d**) CPU-1.3; (**e**) CPU-1.7 and (**f**) CPU-2.0.

The thermal transitions of polymeric materials, such as glass transition temperature (*T*_g_), melting temperature (*T*_m_) and melting enthalpy (*ΔH*_f_), are always be determined by DSC [39]. The DSC thermograms of the COS, PCL, and CPU with different COS content are displayed in Figure 3 and the corresponding thermal transition temperature is listed in Table 2. There was no obvious *T*_g_ observed in the thermogram of COS (Figure 3a), which should be due to the low molecular weight (~3000 g/mol). A broad melting endothermic peak (*T*_m_) around 45–130 °C with Δ*H*_f_ of 52 J/g signified that COS was a noncrystalline polymer [40]. The *T*_g_ of PCL was observed at −58.7 °C (Figure 3b), which had been reported in a previous study [41]. The *T*_m_ observed at ~61.2 °C with Δ*H*_f_ of 61.8 J/g was assigned to the melting transition of crystallized segments, which demonstrated the high crystallinity of PCL. Two glass transition temperatures (*T*_g1_ and *T*_g2_) were observed in the thermograms of CPU films with different COS content (Figure 3c–f). The first glass transition point (*T*_g1_) at a low temperature of ~−20 °C was attributed to the soft segments. Another glass transition area (*T*_g2_) at a high temperature was observed within broad temperatures of from 46 to 64 °C. Because the polarity of urethane (–OCONH–) or/and ureido (–NHCONH–) groups in hard segments is higher than that of ester groups in soft segments, the hard segments are hardly miscible with the soft segments, resulting in micro-phase separation and the appearance of two clear *T*_g_ for soft and hard domains [42]. The broad temperature range (*T*_g2_) probably corresponded to the relaxation of mixed amorphous intermediate phase (soft and hard domains) [43]. In addition, one endothermic peak with broader temperature (*T*_m_) was found (Table 2), and the Δ*H*_f_ increased from 48 to 133 J/g with the increasing content of COS (0~20 wt %) and hard segments (17.5~23.9 wt %) in CPU. The broad endothermic peak should be attributed to the melting transition of COS segments and hard domains. There was no obvious melting endothermic peak of PCL segments in the thermograms, which is probably because the multiple H-bonds between urethane/ureido and ester groups and crosslinking restrict the movement of PCL segments as the crystallization of the soft segment decreases [44].

### 3.3. Thermal Stability

**Figure 4:**
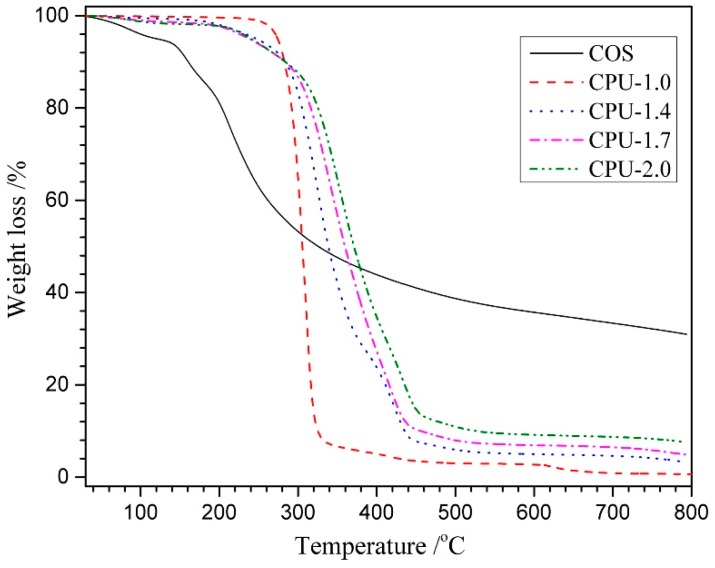
Thermogravimetric analysis (TGA) curves of COS powder and CPU films with different COS content.

The thermal stability of materials is often evaluated by TGA, and from the results, it can deduce the mechanism by which a material loses weight as a result of controlled heating [45]. Figure 4 shows the TGA curves of COS powder and CPU films with different COS content. It could be seen that two consecutive weight loss steps were observed in the COS. The first stage of weight loss (~4.6 wt %) between 30 and 110 °C was responsible for the water loss in COS, indicating its hygroscopic nature. The weight loss of about 45 wt % in the second step with a rapid decomposition between 155 and 330 °C was ascribed to the complex disintegration processes including saccharide rings and macromolecule chains of COS. Approximately 30 wt % remained as residue at the end of the measurement, which indicated that COS had high thermal stability at higher temperature. The thermal degradation of COS, the same as CH, started with the amino groups and formed an unsaturated structure [46]. Only one weight loss step was observed in the curve of pure PU (CPU-1.0), and it showed higher initial decomposition temperature (245 °C) and lower remaining weight (0.6 wt %) compared with COS. With the COS content increasing from 0 to 20 wt % (CPU-1.0~CPU-2.0), the maximum degradation temperature of CPU films increased from 304 to 378 °C, which should be attributed to the increment of chemical crosslinking density in the total structure. Obviously, the lower initial decomposition temperature and higher remaining weight of CPU (CPU-1.4~CPU-2.0) than pure PU (CPU-1.0) were attributed to the COS segments in CPU.

### 3.4. Crystallization Behavior

**Figure 5:**
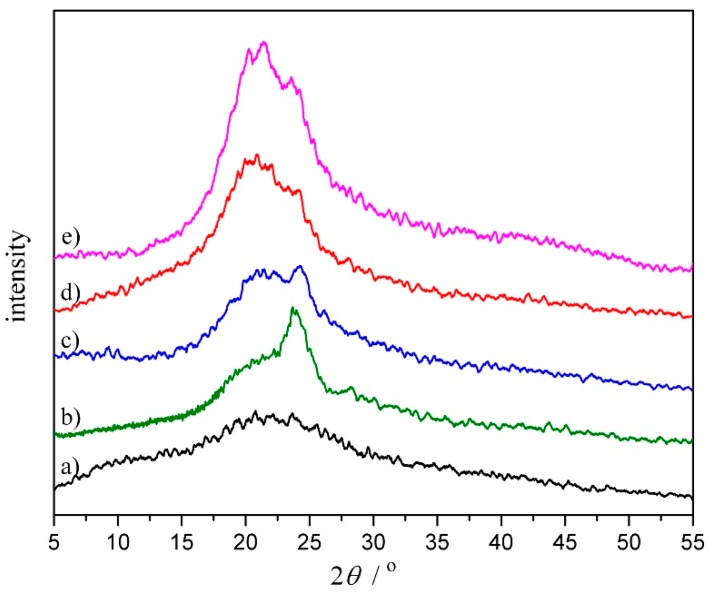
XRD patterns of (**a**) COS powder; (**b**) CPU-1.0; (**c**) CPU-1.4; (**d**) CPU-1.7 and (**e**) CPU-2.0 films.

The crystallization behaviors of the COS powder and CPU films with different COS content were investigated by XRD analysis, and the scattering patterns are shown in Figure 5. A broad diffuse peak appearing in the scattering pattern of COS powder (Figure 5a) signified an amorphous structure, which was consistent with the result of DSC. The pure PU (CPU-1.0, Figure 5b) exhibited a clear diffuse peak with a maximum at 2*θ =* ~23.7°, indicating that pure PU was a hemicrystalline polymer. The crystal composition arised from the crystalline soft domains and uniform-size hard regions formed by the structural symmetry. With the increment of COS content in CPU (CPU-1.4~CPU-2.0, Figure 5c–e), the intensity of diffraction peaks (2*θ =* ~20.7° and 23.7°) increased obviously, which should correspond to the increasing content of uniform-size hard segment. The new diffraction peak (2*θ =* ~20.7°) may be assigned to the ureido formed between COS and the prepolymer, indicating that new crystalline zones were formed. Meanwhile, no sharp diffraction peaks were observed in their scattering patterns. It can be deduced that, with the addition of COS, the CPU forms more crosslinking points which makes it more difficult for COS to react with the prepolymer due to the steric effect, and the unreacted COS is physically mixed with CPU, resulting in slightly blunt peaks in the scattering patterns. 

### 3.5. Mechanical Properties

**Figure 6:**
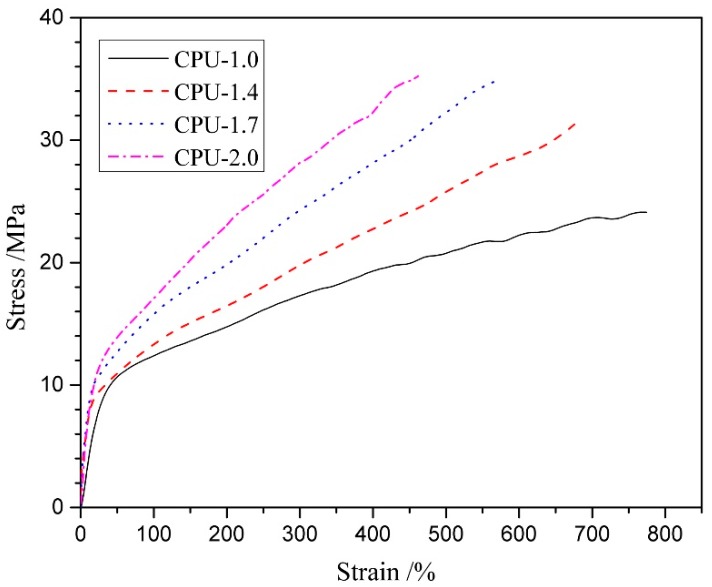
Stress–strain behaviors of CPU films with different COS content.

Mechanical properties are an important quality for biocompatible polymers in soft tissue engineering [47]. The typical stress–strain behaviors of the CPU films with COS content from 0 to 20 wt % are presented in Figure 6, and the characteristic values derived from these curves, including ultimate stress, strain at break, and initial modulus, are shown in Table 3 (pure COS film was too brittle to be obtained because of the low molecular weight). All the films exhibited a similar obvious yield point and manifested as the normal elastomers, which displayed a smooth transition from the elastic to plastic deformation regions in stress–strain behaviors [48]. The pure PU (CPU-1.0) showed an ultimate stress of 24.1 MPa with a strain at break of 774%. The excellent mechanical properties should be related to the uniformity of the hard segments (HBH), which reinforces the hard segments to form hard domains and serves as reinforcing material in a soft segment matrix. In addition, the compact physical-linking network structure, which is formed by the multiple H-bonds existing not only among urethane groups but also between urethane and ester groups, provides an additional energy dissipation mechanism [43]. With the COS content increasing from 9.9 to 20 wt % (CPU-1.4~CPU-2.0), the ultimate stress increased gradually from 30.9 to 35.3 MPa and strain at break decreased from 671% to 462%, as outlined in Table 3. The following two reasons may be explain the results: first, the increased urethane and ureido groups can form more intermolecular hydrogen bonds which give more physical crosslinking points; second, the higher chemical crosslinking density enhances the ultimate stress, especially in a rubbery state. In addition, the initial modulus increased from 25.5 to 53.8 MPa with the COS content increasing from 0 to 20 wt % (Table 3). From these results, it is suggested that the chemical crosslinking density is very important to the mechanical properties of materials and the mechanical properties of CPU can be controlled by adjusting the crosslinking density. 

### 3.6. Surface Hydrophilicity and Swellability

**Figure 7:**
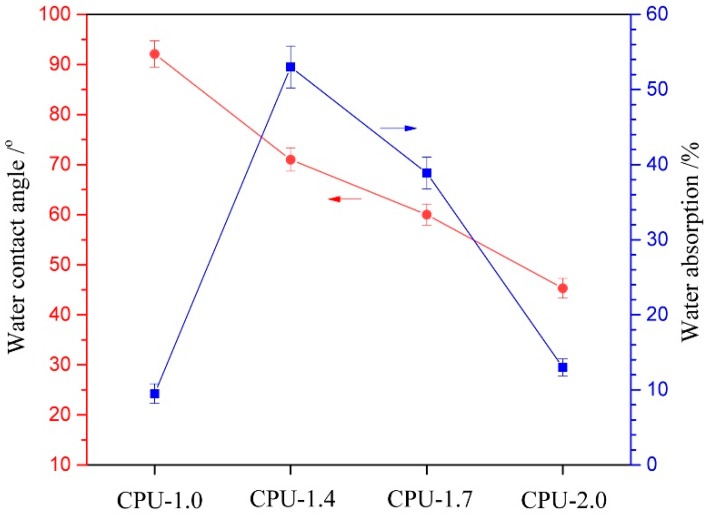
Water contact angle and water absorption of CPU films with different COS content.

The surface hydrophilicity and swellability, which are commonly related to the protein adsorption, platelet adhesion, and biodegradability, are important parameters for biomaterials in many medical applications [49]. The surface hydrophilicity and swellability of the CPU films with different COS content were evaluated by measuring the water contact angle and water absorption, and the results are displayed in Figure 7. The pure PU (CPU-1.0) exhibited a characteristically high water contact angle of 92°, indicating a hydrophobic surface. When the COS content in CPU increased from 9.9 to 20 wt %, the surface hydrophilicity increased gradually with the water contact angle decreasing from 71° to 45.3°, which was ascribed to the introduction of hydrophilic (unreacted) amino groups and hydroxyl groups in COS segments. The result showed that the COS content had outstanding effect on the surface hydrophilicity. The low water absorption (9.5 wt %) of CPU-1.0 indicated that pure PU had a bulk hydrophobic structure, which was consistent with the results for the water contact angle. While CPU-1.4 with lower COS content exhibited a higher water absorption (53 wt %), and with the increment of COS content in CPU (CPU-1.4~CPU-2.0), the water absorption decreased sharply. Obviously, the water absorption is closely related to two factors: hydrophilic chain segment and crosslinking density. The low COS content results in few crosslinking points and the water absorption is mainly affected by hydrophilic COS segments. In addition, the introduction of COS can somewhat destroy the well-defined structure. Thus, water molecules can pass through the film easily, resulting in high water absorption. When the COS content is higher, more crosslinking points are formed, which limits the movement and relaxation of the chains in the films and restricts water to get into the matrix. Consequently, higher crosslinking density can make the films more difficult to swell in water.

### 3.7. In Vitro Degradation

**Figure 8:**
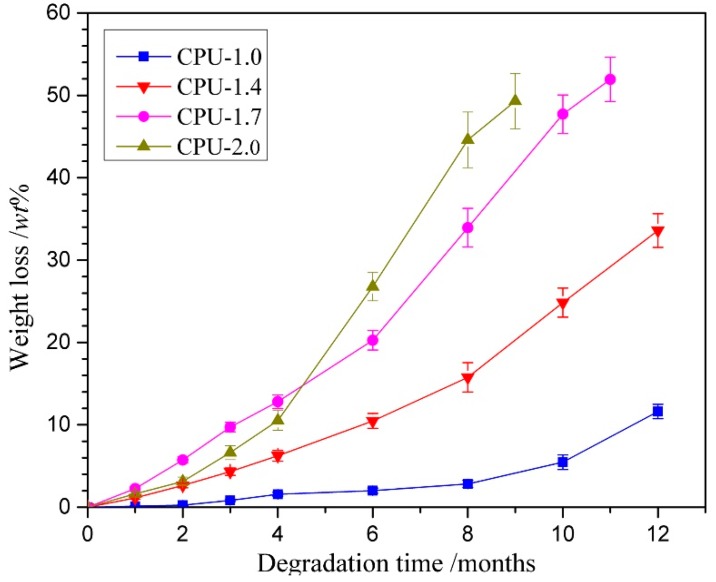
Degradation behaviors of CPU films with different COS content in PBS (pH: 7.4) at 37 ± 0.1 °C.

The degradation behavior has a crucial impact on the performance of implant biomaterials. The degradation behaviors were examined in vitro in PBS at 37 °C, and the percentage weight loss of the CPU films with time are shown in Figure 8. The pure PU (CPU-1.0) exhibited a slow degradation rate with less than 2 wt % after 6 months, and the film had no obvious change except that the transparency decreased. Only 11 wt % weight loss was observed until the end of the test. It is evident that the degradation is mainly caused by hydrolysis of ester groups, the low surface and bulk hydrophilicity and a more compact network structure formed by hydrogen bonds hinder water to approach the ester groups, resulting in slow hydrolytic degradation rate. When the COS was introduced into CPU (CPU-1.4~CPU-2.0), the degradation rate increased obviously after a slow weight loss during two months. The degradation rate increased with the increment of COS content in CPU, which was not in agreement with the results of swellability mentioned above. On the one hand, chemical crosslinked network can somewhat destroy the ordering of polymer structure and reduce the mobility of the chain, which allow the ester groups to be easily exposed to water and result in an increased susceptibility to degradation [38]. On the other hand, the process of degradation of crosslinked materials can be accelerated by blending with polymers susceptible to degradation [50]. In the case of CPU with higher COS content (especially CPU-2.0), the unreacted COS being physically mixed with CPU, as described in XRD, acts as a plasticizer and makes the material more ductile. Then, it was easy for chain scission to take place through hydrolysis of ester bonds. The results are consistent with that of COS-based WPU [16], and manifests that the degradation rate of CPU can be controlled by adjusting the COS content. 

**Figure 9:**
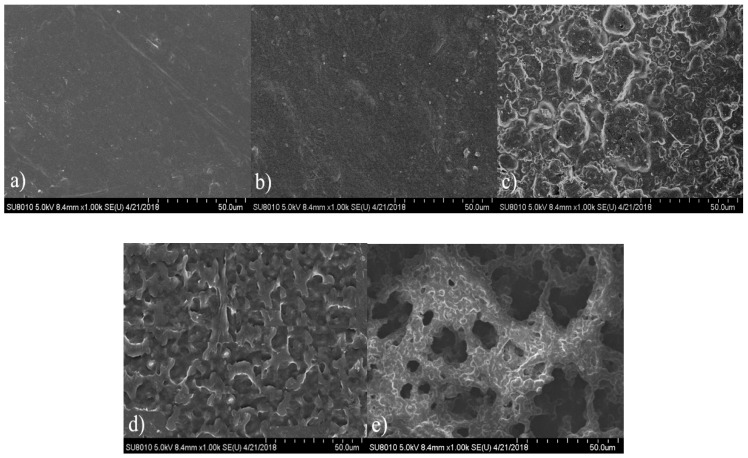
Surface morphologies of CPU-1.4 film in PBS (pH: 7.4) at 37 ± 0.1 °C after (**a**) 0; (**b**) 3; (**c**) 6; (**d**) 10; and (**e**) 12 months’ degradation.

The hydrolytic degradation process can be demonstrated directly by morphological changes in film surface. Figure 9 shows the typical surface morphologies of CPU-1.4 after different degradation periods (predegradation and 3, 6, 10 and 12 months’ postdegradation). The non-degraded film (Figure 9a) was pale-brown semitransparent and exhibited a smooth surface. After three and six months of degradation (Figure 9b,c), the surface became rougher and rougher, and then turned into many irregular hollows at ten months (Figure 9d) which should be due to the loss of unreacted COS. At the end of the measurement, large cavities appeared (Figure 9e), indicating that the film gradually lost its mechanical properties. 

### 3.8. Protein Adsorption

**Figure 10:**
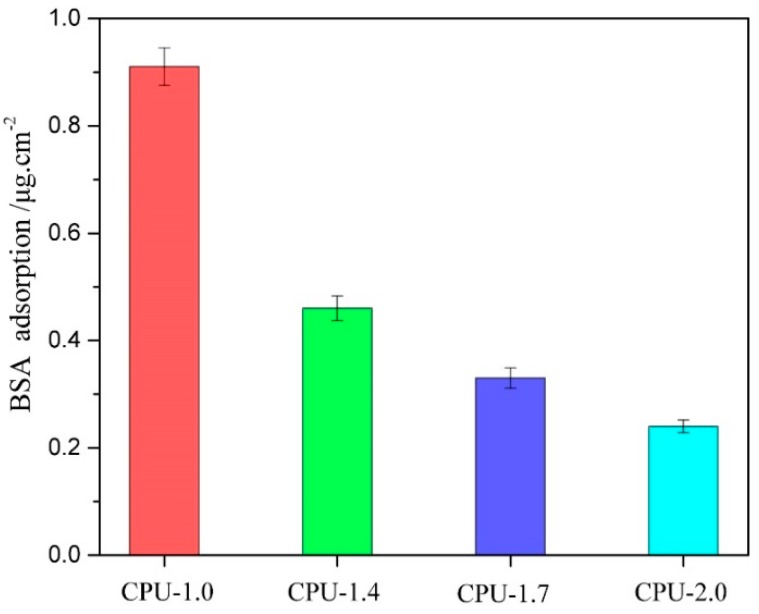
Amount of BSA adsorbed on the surface of CPU films with different COS content at 37 ± 0.5 °C.

One of the most important measurements to evaluate the hemocompatibility of the implantable materials is plasma protein adsorption [51]. The adsorption of BSA on the CPU surfaces was examined to investigate the effects of the COS content on the interaction between the surface and the proteins. The amount of BSA absorbed on the film surface is exhibited in Figure 10. Pure PU (CPU-1.0) exhibited high adsorption of BSA protein (0.91 μg/cm^2^), and the adsorbed amount of protein decreased after the introduction of COS into the films. With the COS content increased from 9.9 to 20 wt % (CPU-1.4~CPU-2.0), the amount of adsorbed protein decreased gradually from 0.43 to 0.24 μg/cm^2^. It was attributed to the hydrophilic amino groups and hydroxyl groups of COS segments on the interface, which could bond with the water molecules to form a hydrated layer and reduce the interaction with protein, leading to a repulsive force to protein. Our experimental data were consistent with the previous reports [32,52,53]: the amount of BSA protein adsorbed on the film surface decreased while the surface hydrophilicity increased. The result is also in accordance with the surface hydrophilicity mentioned above. The lower protein adsorption capacity of CPU films indicated that they had better hemocompatibility than pure PU films.

### 3.9. Platelet Adhesion

**Figure 11:**
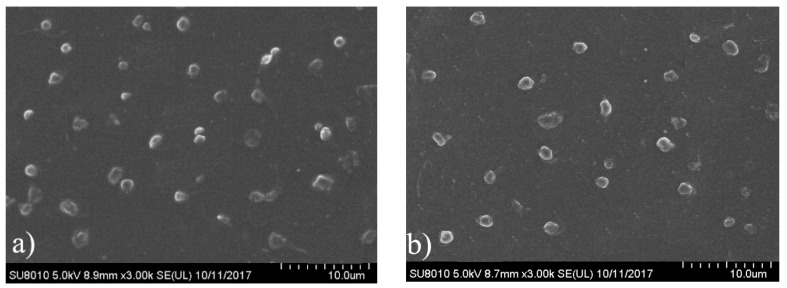

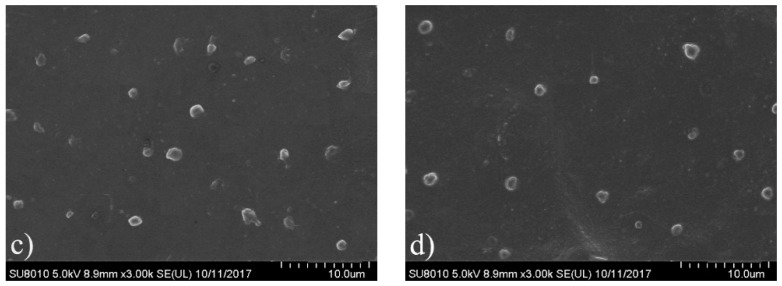
Representative SEM micrographs of platelet adhesion on the surface of (**a**) CPU-1.0; (**b**) CPU-1.4; (**c**) CPU-1.7 and (**d**) CPU-2.0 films.

Platelet adhesion on the film surface is another important test for evaluation of the hemocompatibility of the implantable materials. Obviously, the less interaction between the platelet and the material surface means the lower probability of thrombus [15]. The morphologies of the platelets adherent on the surfaces of CPU films were assessed by SEM observation, and the representative micrographs are given in Figure 11. The surface distribution of platelets on pure PU surface (CPU-1.0, Figure 11a) was non-random and aggregated to some extent, presenting the highly activated state. As shown in the Figure 11b–d, the platelet adhesion was obviously reduced on the surface of COS-based CPU films. The amount of adhesive platelet on the surface decreased gradually with the increment of COS content, and no obvious gathering of platelets was found, which proved a better anti-platelet adhesion surface [54]. One possible explanation is that the hydrophilic surface of films suppresses platelet adhesion. The trend of platelet adhesion was consistent with that of protein adsorption.

## 4. Conclusions

In this work, a series of biodegradable COS-based PUs (CPU) with uniform-size hard segments were prepared in DMF via the conventional two-step method, in which COS was employed as a chain extender. The corresponding films were obtained by the solvent evaporation method. The chemical structure was characterized by FT-IR, and the influence of COS content in CPU on the physicochemical properties and hemocompatibility was extensively researched. The thermal stability studies indicated that the CPU films had a lower initial decomposition temperature and higher maximum decomposition temperature than pure PU (CPU-1.0) film. The ultimate stress, initial modulus, and surface hydrophilicity increased with the increment of COS content, and, due to the increment of crosslinking density, the strain at break and water absorption decreased. In vitro degradation studies showed that the degradation rate increased with the increasing content of COS in CPU, demonstrating that the degradation rate could be controlled by adjusting COS content. The surface hemocompatibility was evaluated by protein adsorption and platelet adhesion tests, and the results demonstrated that the CPU surface had improved resistance to protein adsorption and possessed good resistance to platelet adhesion. The slow degradation and good hemocompatibility of the CPUs display great potential in blood-contacting devices. Moreover, many active amino and hydroxyl groups contained in the structure of CPU could carry out further modification, which made it an excellent candidate for wide application in biomedical field. 

## Figures and Tables

**Table 1:** The basic formulations and chitooligosaccharide (COS) content of chitooligosaccharide-based polyurethane (CPU).

Samples	PCL/g	HBH/g	COS/g	COS Content/wt % *	*n*_-OH_:*n*_–NCO_:*n*_–NH2_ **
CPU-1.0	8.0	1.70	0	0	1:1:0
CPU-1.4	8.0	2.39	1.14	9.89	1:1.4:0.8
CPU-1.7	8.0	2.90	2.0	15.5	1:1.7:1.4
CPU-2.0	8.0	3.41	2.86	20.0	1:2.0:2.0

* COS content in CPU; ** the molar ratio of –OH in PCL, –NCO in HBH and –NH_2_ in COS.

**Table 2:** The thermal transition temperatures of COS, PCL, and CPUs.

Samples	COS	PCL	CPU-1.0	CPU-1.4	CPU-1.7	CPU-2.0
*T*_g1_ (^o^C)	-	−58.7	−17.4	−21.0	−18.3	−20.2
*T*_g2_ (^o^C)	-	-	48~63	46~61	46~62.5	45.5~63
*T*_m_ (^o^C)	45~130	61.2	90~118	83~123	82~125	80~129
Δ*H*_f_ (J/g)	52	61.8	48.2	78.3	108.9	133.4

**Table 3:** Mechanical properties of CPU films.

Films	Strain at Break (%)	Ultimate Stress (MPa)	Yield Stress (MPa)	Yield Strain (%)	Initial Modulus (MPa)
CPU-1.0	774 ± 17	24.1 ± 2.2	10.6 ± 1.6	41.3 ± 3.5	25.5
CPU-1.4	671 ± 15	30.9 ± 1.8	9.3 ± 1.1	22.6 ± 1.2	41.2
CPU-1.7	569 ± 15	34.9 ± 1.6	10.2 ± 1.1	19.7 ± 1.0	51.7
CPU-2.0	462 ± 13	35.3 ± 1.6	11.9 ± 1.2	22.1 ± 1.3	53.8
